# Knowledge, Attitudes, and Practices on Rocky Mountain Spotted Fever among Physicians in a Highly Endemic Region—Mexicali, Mexico

**DOI:** 10.4269/ajtmh.21-1017

**Published:** 2022-08-22

**Authors:** Nicolette Bestul, Rosario Padilla, Tania Montaño, Adriana Márquez, Maria Fierro, Oscar E. Zazueta, Paige A. Armstrong

**Affiliations:** ^1^ Rickettsial Zoonoses Branch, Division of Vector-Borne Diseases, Centers for Disease Control and Prevention, Atlanta, Georgia;; ^2^ Secretaría de Salud de Baja California, Mexicali, Baja California, México;; ^3^ Imperial County Health Department, El Centro, CA

## Abstract

Rocky Mountain spotted fever (RMSF) is a potentially fatal tickborne disease caused by the bacterium, *Rickettsia rickettsii* and transmitted primarily by the brown dog tick (*Rhipicephalus sanguineus*) in the southwestern United States and Mexico. RMSF can be rapidly fatal if not treated early with doxycycline, making healthcare worker awareness and education critical to reduce morbidity and mortality. During 2008–2019, Mexicali experienced a RMSF epidemic with 779 confirmed cases, and an 11-year case-fatality rate of 18% (*N* = 140). A cross-sectional study was conducted with 290 physicians and physicians-in-training across 12 medical facilities in Mexicali. They were asked to complete a 23-item questionnaire to assess knowledge, attitudes, and practices for clinical, epidemiologic, and preventive aspects of RMSF. Half of participants were female, the largest age group was aged 25 to 44 (47%), and median time in practice was 6 years (interquartile rate: 1–21.5). Less than half (48%) surveyed were confident where diagnostic testing could be performed, and two-thirds did not regularly order serology (67%) or molecular diagnostic (66%) tests for RMSF when a patient presented with fever. Sixty-four percent knew doxycycline as first-line treatment of children < 8 years with suspected RMSF. When comparing healthcare workers with < 6 years of experience to those with ≥ 6 years, more experience was associated with greater confidence in where to have diagnostic testing performed (prevalence odds ratio [prevalence odds ratios [pOR]] = 2.3; *P* = 0.004), and frequency of ordering laboratory tests (serology, pOR = 3.3; *P* = 0.002; polymerase chain reaction, pOR = 3.9; *P* = 0.001). Continued education, including information on diagnostic testing is key to reducing morbidity and mortality from RMSF.

## INTRODUCTION

Rocky Mountain spotted fever (RMSF) is a potentially fatal tickborne disease (TBD) caused by *Rickettsia rickettsii* that, if left untreated, can cause widespread vasculitis resulting in multiple organ failure and death.^1^ RMSF has nonspecific early symptoms including fever and headaches with most patients developing a rash at some point in the illness.^2^ However, during 2009–2019 in Mexicali, Mexico, only 43% of laboratory-confirmed cases reported rash presentation.^3^ The delay in appearance or absence of a rash can make an RMSF diagnosis challenging. Early treatment with doxycycline can prevent severe outcomes in all age groups. Doxycycline is most effective if started within the first 55 days of illness and should not be delayed awaiting confirmatory diagnostic results.^1^ Multiple tick species transmit the bacteria, with regional differences in the most abundant vector. RMSF transmission is most often associated with the eastern, central, and western United States with the American dog tick (*Dermacentor variabilis*).^1^ However, in Arizona and northern Mexico, the brown dog tick (*Rhipicephalus sanguineus* sensu lato) is the predominant vector.^4^^–^^8^ The brown dog tick can spend all life stages on dogs, leading to heavy levels of infestation, which has been associated with an increased incidence of human cases.^9^^,^^10^

Since 2008, a reemergence of RMSF has been ongoing in Mexicali, Mexico, a metropolitan area of slightly over one million people in the Baja California state, immediately south of California, United States.^11^^,^^12^ The incidence and absolute case counts are much higher than had been seen previously. As of 2019, at least 4,290 people were suspected to have had RMSF, with 779 confirmed cases and an 11-year case-fatality rate of 18% (*N* = 140) during 2008–2019.^3^^,^^13^^–^^15^ An epidemiologic emergency for RMSF was declared by the Mexican Ministry of Health in 2015 in response to this epidemic, and it was updated in 2018.^16^

Previous research on vector-borne diseases indicated that people’s participation in preventive measures is related to their knowledge, attitudes, and practices (KAPs) regarding transmission and disease symptoms.^17^^–^^19^ Low levels of KAPs have also been associated with low levels of participation in prevention practices.^20^ This study aims to evaluate KAPs for RMSF among physicians and physicians-in-training in Mexicali, Mexico, to improve communication and guidance to healthcare providers about RMSF and other TBDs.

## MATERIALS AND METHODS

### Setting and study population.

Mexicali is the capital of the northern state of Baja California, Mexico. It has a population of around one million persons, and it is the immediate border with Imperial County in California.^11^^,^^12^ Five primary healthcare providers serve individuals in Mexicali: Institute for Social Security and Services for State Workers, Mexico Social Security Institute, Institute for Social Security and Services for Government and Local Workers of the State of Baja California, Institute of Public Health Services of Baja California (ISESALUD), and the private sector (e.g., outpatient clinics adjacent to private pharmacies for mild cases and private hospitals for severe cases). Healthcare workers, including medical students, interns, residents, and attending physicians, including physicians working at pharmacies, were all eligible for inclusion. All public hospitals in the city were visited, and individuals were invited to attend a grand rounds lecture on RMSF given by physicians and public health professionals. Prior to the lecture and before any discussion of the content, attendants were provided with a survey to complete independently (Supplemental Appendix 1). A limited number of questionnaires were given partway through the lecture when participants arrived late. Primary care physicians working in private pharmacies were recruited by visiting pharmacies and surveying the physician working at the time. Participation in the study was voluntary and verbal consent was obtained from each healthcare worker before the lecture. The institutional review board (IRB) determined written consent was not needed because no identifiable information was being collected and obtaining signatures from every physician would be overly burdensome given the number of physicians completing the survey at a given time. Questionnaires were administered at 12 medical facilities between January and July 2019, representing all five healthcare sectors.

### Survey tool.

The questionnaire was divided into four sections: general information (six items), attitudes and perception (five items), individual practices (six items), and knowledge (12 items). The general information questions collected basic demographic data (age and sex), as well as the location and setting of their practice, their specialty, and years practicing. Attitude and perception questions assessed the healthcare workers’ confidence regarding RMSF knowledge, diagnosis, and testing. This section also showed how strongly they believed that TBDs posed a serious health risk to their community. The individual practice questions assessed how often each healthcare worker discussed risk factors and other RMSF information with patients, as well as how frequently they diagnosed a case. Finally, the knowledge questions assessed understanding of RMSF clinical presentation, recommended treatment, complications, and prevention methods. Knowledge was assessed with 12 multiple-choice and true/false questions, with a total possible of 19 points. The questionnaire was developed using previous infectious disease KAP surveys as guides but was reworked for RMSF in Mexicali. Additionally, the survey was piloted among 10 physicians prior to full dissemination.

### Analysis.

Data were collected on paper forms and entered into Epi Info 7 (CDC, Atlanta, GA). Data were then exported and analyzed using SAS 9.4 (Cary, NC). Demographic data and basic characterizations for each of the three sections were presented as counts and proportions. Years practicing was presented as a categorical variable grouped in 5-year ranges. When calculating crude prevalence odds ratios (pOR) and *P* values of the three KAP categories, the results were stratified by < 6 and ≥ 6 years of experience because 6 years was the median. Answers given using a Likert scale were grouped into binary categories; “Not Confident” (inclusive of “not confident” and “somewhat confident”) and “Confident” (inclusive of “confident” and “very confident”); “Disagree/Neutral” (inclusive of “strongly disagree”, “disagree”, and “I do not agree or disagree”) and “Agree” (inclusive of “agree” and “strongly agree”); “Less often” (inclusive of “rarely” and “sometimes”) and “More often” (inclusive of “frequently” and “always”). Differences were compared using Fisher’s exact test for small sample sizes or χ^2^ test for large sample sizes, and two-sided statistical tests were considered significant when the *P* value was ≤ 0.05. Finally, a mean knowledge score was calculated, and a one-way analysis of variance test was run to determine whether there was a difference between different years of experience when years of experience was not stratified. Additionally, an independent group *t* test was run to look at differences between the stratified categories of < 6 and ≥ 6 years of experience.

### Ethics.

The protocol underwent review and was approved by the local IRB at the Mexicali General Hospital. CDC’s Human Research Protection Office also reviewed, and it was determined to be nonresearch activity under 45 CFR 46.102(d), and therefore further IRB review was not required.

## RESULTS

A total of 290 healthcare workers completed the questionnaire. About half of the participants were female (*N* = 136, 47%). The largest age group was 25 to 44 years old (*N* = 134, 46%). The largest proportion reported less than 5 years of medical practice (*N* = 96, 33%), but these data were missing in 28% (*N* = 80) of responses. More than half of those surveyed reported working in a hospital (*N* = 148, 51%), and most were general practitioners or did not declare a specialty (*N* = 234, 81%) (Table 1).

**Table 1:** Demographic and practice information for physicians and physicians-in-training participating in knowledge, attitudes, and practices survey—Mexicali, Mexico

	Surveys, *N* = 290	
	*n*	%
Sex		
Male	135	46.6
Female	136	46.9
Missing	19	6.6
Years practicing		
< 5	96	33.1
5–9	32	11.0
10–14	15	5.2
15–19	12	4.1
20–24	7	2.4
25–29	11	3.8
30–34	16	5.5
≥ 35	21	7.2
Missing	80	27.6
Age group		
< 25	79	27.2
25–44	134	46.2
45–64	58	20.0
≥ 65	16	5.5
Missing	3	1.0
Medical specialty		
Yes	41	14.1
Type of specialty		
None/missing	234	80.7
Internal medicine	6	2.1
Infectious diseases	1	0.3
Emergency physician	4	1.4
Family medicine	10	3.5
Pediatrics	7	2.4
Other	28	9.7
Practice setting		
Missing	15	5.2
Hospital	148	51.0
Clinic	77	26.6
Pharmacy	15	5.2
Other	35	12.1

Attitudes and perceptions were asked regarding up-to-date knowledge on RMSF, clinical diagnosis, diagnostic testing, and risk to their community. Across all questions, the most common selection was confident with 515 (44%) of all responses falling in that category. When combined with very confident responses, they accounted for 624 (54%) of all responses. Responders were most confident in selecting the appropriate diagnostic test (*N* = 171, 59%; confident and very confident), and least confident in where the diagnostic testing could be performed (*N* = 139, 48%; not confident, somewhat confident). The most common reason provided for lacking confidence in where to have diagnostic testing performed was they were unsure of the location where testing is available (*N* = 114, 39%), and unsure how to order the correct test (*N* = 72, 25%). Nearly all (*N* = 260, 90%) participants agreed that TBDs were a serious risk for individuals in their community (Table 2).

**Table 2:** Attitudes, perceptions, and individual practices reported by healthcare workers in Mexicali, Mexico (*N* = 290)

Attitudes and perceptions	Not confident		Somewhat confident		Confident		Very confident		Missing			
How confident are you …	*n*	%	*n*	%	*n*	%	*n*	%	*n*	%	*n*	%
On up-to-date knowledge on RMSF?	14	4.8	107	36.9	136	46.9	25	8.6	8	2.8		
Diagnosing on clinical symptoms alone?	24	8.3	107	36.9	132	45.5	23	7.9	4	1.4		
Submitting the type of diagnostic tests for a suspect RMSF case?	21	7.2	92	31.7	140	48.3	31	10.7	6	2.1		
Where to have diagnostic testing performed for a suspect RMSF case?	44	15.2	95	32.8	107	36.9	30	10.3	14	4.8		

			I am not sure how to get the results		I am not sure how to order the correct testing		I am not sure where testing can be performed		Other					
If not confident, why?			37	12.8	72	24.8	114	39.3	17	5.8				

	Strongly disagree		Disagree		I do not agree or disagree		Agree		Strongly agree		Missing	
Tickborne diseases represent a serious risk for my community	6	2.1	5	1.7	6	2.1	88	30.3	172	59.3	13	4.5

Individual Practices	Rarely		Sometimes		Frequently		Always		Does not apply		Missing	
If a patient presents with a febrile illness, how often do you												
Ask if a tick bite in the past 2 weeks	19	6.6	46	15.9	77	26.6	132	45.5	5	1.7	11	3.8
Ask about ticks in home or on pets	10	3.5	27	9.3	91	31.4	151	52.1	–	–	11	3.8
Inquire about dog ownership	8	2.8	18	6.2	75	25.9	178	61.4	–	–	11	3.8
Order one serology test for RMSF	118	40.7	76	26.2	30	10.3	26	9.0	25	8.6	15	5.2
Order PCR to test for RMSF	135	46.6	56	19.3	26	9.0	24	8.3	29	10.0	20	6.9
If a patient asks about RMSF or other tickborne illness, how often do you												
Provide with educational materials	74	25.5	57	19.7	57	19.7	53	18.3	24	8.3	25	8.6
Discuss risk factors	8	2.8	32	11.0	88	30.3	134	46.2	8	2.8	20	6.9
Discuss preventative measures	8	2.8	19	6.6	78	26.9	159	54.8	7	2.4	19	6.6

	Yes		No		Missing							
Diagnosed RMSF case in last year?	186	64.1	81	27.9	23	7.9						

			< 3		3–4		5+		None/missing					
How many?			50	17.2	20	6.9	6	2.1	214	73.8				

	Rarely		Sometimes		Frequently		Always		Does not apply		Missing	
Do you report a diagnosed RMSF case to the health department?	12	4.1	3	1.0	26	9.0	105	36.2	96	33.1	48	16.6

PCR = polymerase chain reaction; RMSF = Rocky Mountain spotted fever.

When inquired about practices, healthcare workers reported *always* (*N* = 461, 53%) or *frequently* (*N* = 243, 28%) asking patients presenting with fever about risk factors for RMSF (i.e., tick bite, ticks in home or on pets, dog ownership). Breaking these down further, asking about a recent tick bite was reported as always 45% (*N* = 132) of the time. Asking about the presence of ticks in home or on pets was reported as always 52% (*N* = 151) of the time. Inquiries about dog ownership were reported as always 61% (*N* = 178) of the time. However, assessment of testing practices showed that healthcare workers infrequently ordered diagnostics. Nearly half of the participants reported rarely ordering polymerase chain reaction (PCR; *N* = 135, 47%) and another 56 (19%) said they ordered it sometimes. Serology was ordered equally infrequently with 118 (41%) reported they ordered it rarely, and 76 (26%) sometimes. When a patient asked about RMSF or other TBD, the largest proportion of healthcare workers reported always discussing risk factors (*N* = 134, 46%) and preventative measures (*N* = 159, 55%), but not the provision of educational material (*N* = 53, 18%). More than half of the healthcare workers reported diagnosing a case of RMSF in the past year (*N* = 186, 64%). Of the 146 for which we have information, 72% (*N* = 105) always reportedly submitted a case to ISESALUD (Table 2).

**Table 3:** Knowledge assessment of healthcare workers in Mexicali, Mexico

Questions (correct answers are shown)	Surveys *N* = 290	
	*n*	%
What percent of patients with RMSF report a history of tick bite?*		
50%	78	26.9
What is the incubation period (time from tick bite to onset of symptoms) for RMSF?†		
3–14 days	165	56.9
Treatment should not be started until the rash develops.		
False	276	95.17
Rash typically develops between days 2–5 of illness.		
True	128	44.14
Rash develops in approximately 90% of people but may appear after the 5th day of illness.		
True	168	57.93
Antibiotic therapy should be initiated‡		
As soon as you suspect RMSF	240	82.76
The antibiotic of choice for treatment of RMSF in children ≤ 8 is§		
Doxycycline	185	63.79
The antibiotic of choice for treatment of RMSF in adults and children > 8 years old is§		
Doxycycline	261	90
When is therapy for RMSF most effective?¶		
In the first 5 days from the start of the symptoms	246	84.83
The fatality rate of untreated RMSF is#		
>50%	207	71.38
Which of the following severe manifestations are associated with RMSF?**††		
Gangrene requiring amputation	106	36.55
Cerebral edema and altered mental status	118	40.69
Severe thrombocytopenia	229	78.97
Pulmonary edema and acute respiratory distress syndrome (ARDS)	98	33.79
Over what period of time do the sequela listed above manifest?##		
5–7 days from the start of symptoms	82	28.28
Current recommendations to prevent RMSF††		
Monitor closely for fever, or other symptoms for 2 weeks after a known tick bite.	187	64.48
Avoiding outdoor activities and limiting dog ownership‡‡	251	86.55
Careful inspection for, detection of, and removal of ticks from pets or persons before or soon after they attach.	219	75.52
Use of tick repellent when tick exposure is a possibility and use of tick repellent or tick-killing substances on pets.	179	61.72
Applying to pesticide to yards and homes.	188	64.83

PCR = polymerase chain reaction; RMSF = Rocky Mountain spotted fever.

Other answers were as follows:

* 90% and 25%.

† 24–36 hours and 14–21 days.

‡ After being confirmed in laboratory diagnosis, after the appearance of dermatological lesions, and none of the above.

§ Azithromycin, chloramphenicol, or trimethoprim-sulfamethoxazole. For children, the second most common answer was chloramphenicol with 12%.

¶ Within days 7–10 of the onset of symptoms; in the first 2 weeks from the start of symptoms; and antibiotic therapy timing is not important.

#Zero, < 10%, and 20–25%.

**Percent that responded true for each individual option.

## 10 days from the start of symptoms; they are very infrequent and the appearance time is not well established.

†† More than one answer could be selected.

‡‡ Correct answer was false.

The median knowledge score was 13 (range: 2–18). Healthcare workers identified key presenting symptoms (fever, pain, and rash) as present *always* or *frequently* a majority of the time (*N* = 252, 87%; *N* = 236, 81%; and *N* = 213, 73% for each symptom, respectively). Almost all responders knew that treatment should not wait until a rash develops (*N* = 276, 95%). A majority also understood that antibiotics should be started as soon as RMSF is suspected (*N* = 240, 83%), and antibiotics are most effective within the first 5 days after the start of the symptoms (*N* = 246, 85%). Additionally, whereas nearly all healthcare workers knew doxycycline is the treatment of choice in patients 8 years and older (*N* = 261, 90%), fewer selected doxycycline as the appropriate treatment in patients less than 8 years of age (*N* = 185, 64%). Even though a majority knew that the fatality rate of untreated RMSF was > 50% (*N* = 207, 71%), some of the severe manifestations were much less well known with 41% or less associating the following with RMSF: gangrene requiring amputation (*N* = 106, 37%), cerebral edema and altered mental status (*N* = 118, 41%), and pulmonary edema and acute respiratory distress syndrome (*N* = 98, 34%). However, most did know that severe thrombocytopenia (*N* = 229, 79%) was associated with RMSF. The questions with the lowest overall correct scores were “What percent of patients with RMSF report a history of tick bite?” (*N* = 78, 27%) and “Over what period of time do the sequela listed above manifest?” (*N* = 82, 28%) (Table 3).

When stratified by years practicing, healthcare workers with 6 years or more of practice had higher odds of feeling confident in the location to have testing performed (pOR = 2.3; *P* = 0.004) and, although not statistically significant, the appropriate type of diagnostic test to order (pOR = 1.7; *P* = 0.07). They also had higher odds of asking patients about tick exposure (pOR = 2.0; *P* = 0.05) and order serology (pOR = 3.3; *P* = 0.002) or PCR (pOR = 3.9; *P* = 0.001). However, they had lower odds of discussing risk factors (pOR = 0.2; *P* = 0.001) compared with those with less than 6 years of experience. Differences in knowledge scores were only statistically significant (*P* ≤ 0.05) when comparing the group with the highest average score, those with 10 to 24 years practicing (score = 14.03), to the group with the lowest average score, those with more than 25 years of experience (score = 11.46). Few individual question scores were statistically significantly different when looking at them stratified by those practicing less than 6 years versus those practicing 6 years or more. The latter, however, had lower odds of knowing avoiding outdoor activities and limiting dog ownership were not current recommendations to prevent RMSF (pOR = 0.3; *P* = 0.003). We performed the analysis by age of provider and did not find additional statistically significant differences (Table 4).

**Table 4:** Knowledge, attitudes, and practices (KAP) results stratified by years practicing by healthcare workers in Mexicali, Mexico

	All respondents *N* = 210¥		< 6 years *N* = 102		≥ 6 years *N* = 108		pOR (95% CI)	*P* value
Attitudes and perceptions	*n*	%	*n*	%	*n*	%		
Diagnosing								
Not confident	87	41.8	46	45.1	41	38.7	1.3 (0.7–2.3)	0.3485
Confident	121	58.2	56	54.9	65	61.3		
Types of diagnostic tests								
Not confident	71	34.5	41	40.6	30	28.6	1.7 (1.0–3.1)	0.0706
Confident	135	65.5	60	59.4	75	71.4		
Where to do diagnostic tests								
Not confident	99	49.3	59	59.6	40	39.2	2.3 (1.3–4.0)	0.0041*
Confident	102	50.7	40	40.4	62	60.8		
If not confident, why?								
Not sure how to get the results	26	12.4	14	13.7	12	11.1	0.8 (0.3–1.8)	0.5660
Not sure how to order the correct testing	46	21.9	25	24.5	21	19.4	0.7 (0.4–1.4)	0.3759
Not sure where testing can be performed	84	40	52	51.0	32	29.6	0.4 (0.2–0.7)	0.0018*
Tickborne diseases are a serious risk								
Disagree/neutral	14	6.9	6	6.0	8	7.8	0.8 (0.3–2.2)	0.6071
Agree	188	93.1	94	94.0	94	92.2		

Individual practices	*n*	%	*n*	%	*n*	%		
Ask if person had tick bite in past 2 weeks								
Less often	47	23.3	29	29.3	18	17.5	2.0 (1.0–3.8)	0.0489*
More often	155	76.7	70	70.7	85	82.5		
Ask about ticks in home or on pets								
Less often	26	12.7	15	15.0	11	10.5	1.5 (0.7–3.5)	0.3329
More often	179	87.3	85	85.0	94	89.5		
Order one serology test for RMSF								
Less often	141	77.5	81	87.1	60	67.4	3.3 (1.5–6.9)	0.0020*
More often	41	22.5	12	12.9	29	32.6		
Order PCR to test for RMSF								
Less often	137	79.7	79	89.8	58	69.0	3.9 (1.7–9.0)	0.0012*
More often	35	20.3	9	10.2	26	31.0		
Discuss risk factors								
Less often	25	13.0	4	4.2	21	21.6	0.2 (0.1–0.5)	0.0012*
More often	167	87.0	91	95.8	76	78.4		
Discuss preventative measures								
Less often	15	7.8	5	5.3	10	10.2	0.5 (0.2–1.5)	0.2076
More often	178	92.2	90	94.7	88	89.8		
Diagnosed RMSF case in last year?								
Yes	136	69.4	67	70.5	69	68.3	1.1 (0.6–2.0)	0.7373
No	60	30.6	28	29.5	32	31.7		
How many?								
< 3	34	60.7	16	64	18	58.1	Ref	Ref
3–4	16	28.6	8	32	8	25.8	1.1 (0.3–3.7)	0.8461
≥ 5	6	10.7	1	4	5	16.1	0.2 (0.02–2.1)	0.1938

Knowledge	Mean	IQR	Mean	IQR	Mean	IQR	*t* value	*P* value
Median score	12.2	11.7–12.6	12.5	11.9–13.2	12.5	11.8–13.1	0.45	0.6634

CI = confidence interval; IQR = interquartile range; PCR = polymerase chain reaction; pOR = prevalence odds ratio; RMSF = Rocky Mountain spotted fever. Data in table are reported as n, % unless otherwise indicated. Categories might not sum to 100% if data points were unknown, or if categories were not mutually exclusive.

¥ Only includes samples with a known value for years practicing.

* Indicates statistical significance at *P* ≤ 0.05.

## DISCUSSION

Assessment of healthcare workers’ practices in a highly endemic region for RMSF is key to identifying gaps and ensuring best practices to improve patient outcomes. KAP surveys are a valuable way of assessing gaps and targeting education and interventions. In this survey, we saw that nearly all contributing healthcare workers recognized TBDs are important in their city, with many having reported treating a case in the past year. Although the importance of TBDs was known, the gaps recognized emphasized the need to have continued incorporation of RMSF education in the medical school curriculum, and to explore other avenues to provide continuing and supplemental education to healthcare workers already in practice. Further, because cases in Mexicali remain high, reporting them to the health departments is crucial to understanding the epidemiology of the current epidemic. Most (*N* = 105, 72%) of the physicians that diagnosed a case in the past year always reported these cases to the health department, but this still leaves almost 30% of physicians not consistently reporting. Without these reports, the burden of RMSF may be underestimated in Mexicali, and clusters of cases could go unidentified.

Early diagnosis and appropriate treatment across all age groups are critical to reduce mortality. Multiple studies have been conducted to understand the role that healthcare workers play in outcomes. A study in Arizona from 2002 to 2011 of two tribal communities showed that even when patients presented for care early, those who received doxycycline later (after 5 days) had a greater likelihood of fatal outcome.^21^ This study also found that healthcare workers often failed to include RMSF in the differential diagnosis initially and that they were more likely to rely on common clinical presentations, such as rash, or diagnostic results to decide to initiate treatment.^21^ Additionally, a study performed in 2009 in Tennessee, assessed healthcare workers’ knowledge, attitudes, and practices and found a large proportion of respondents also reported waiting for a rash to provide treatment and thought that treatment could be delayed up to 2 weeks and remain effective.^22^ Participants in our Mexicali study were familiar with recommendations on when to treat and knew they should not wait for a rash or test results to confirm the diagnosis. The nonspecific nature of RMSF symptoms makes this knowledge important, and, although many responders knew rash to be a key symptom, it can often present later in the infection or not at all. Our findings also showed great overall knowledge of doxycycline treatment. However, gaps existed in age-based treatment guidelines. This has been seen in a variety of regions. A cross-sectional survey of physicians in 2016 in Sonora, Mexico, showed that healthcare workers lacked knowledge that doxycycline is the first-choice treatment of children under 8 years, the correct time to initiate doxycycline, and the case-fatality rate of untreated RMSF.^23^ The 2009 Tennessee study also found that a high percentage of those surveyed were unaware that doxycycline is the treatment of choice for children under 8 years.^22^ One reason why we may be seeing a lower percentage of proper treatment among children was previous concerns regarding dental staining and enamel hypoplasia due to doxycycline. However, no evidence of harmful effects has been shown with the use of short-term doxycycline treatment.^24^ Yet misconceptions about treatment persist, as was seen in our Mexicali study, where 90% (*N* = 261) of healthcare workers knew doxycycline was the recommended treatment in ≥ 8 years of age but only 64% (*N* = 185) in < 8 years of age. This finding did not differ significantly among those practicing for less than 6 years (*N* = 66, 72%) compared with 6 years or more (*N* = 75, 77%). Proper antibiotic treatment could prove to be essential in limiting the impact of RMSF in Mexicali. Pediatric RMSF cases have historically had the highest reported case-fatality rate. This Mexicali epidemic has had a nearly 37% case-fatality rate among PCR-positive pediatric cases.^3^ Continued emphasis on proper treatment recommendations in children is key in clinical education especially given that treatment with other antibiotics is also shown to be associated with increased mortality.^1^

Whereas the decision to treat is based on clinical diagnosis, diagnostic testing remains important for confirmation and to inform surveillance and risk stratification in a community.^3^ Although diagnostic testing may not inform the current treatment decision, it provides invaluable evidence about the incidence and prevalence of certain diseases in a community. This understanding of the pathogens circulating in the community can then help direct healthcare workers when making future treatment decisions for an individual presenting with undifferentiated febrile illness. These data can also allow for monitoring of trends, either across age categories or locations in the city, allowing for targeted interventions and concerted prevention campaigns. A sizable portion of those surveyed in this study was not familiar with where to have testing performed. This presents another opportunity for healthcare worker education.

Time in practice was associated with both confidence and certain practice patterns. Those with 6 years or more in practice reported having significantly more confidence in knowing where to have diagnostic testing performed, asking about the history of a tick bite, and ordered diagnostic testing more frequently. Those with less than 6 years of experience were significantly more likely to discuss risk factors for acquiring RMSF infection and were more knowledgeable regarding the timeline of when sequela would manifest. The overall consistency in the counseling of preventative measures and risk factors was high, especially among those at the start of their careers, but there is variation in the knowledge of appropriate preventive measures, laboratory diagnostics, and treatment guidelines. Continuing to incorporate education on RMSF into the healthcare curriculum is important to ensure the next generation of healthcare workers are prepared to combat this ongoing public health threat.

There were limitations in this study. For participants from hospitals, we recruited healthcare workers at a lecture on RMSF, so individuals more interested in the topic may have been more likely to attend or had additional, preexisting knowledge on the topic. This may have falsely elevated knowledge scores. Additionally, healthcare workers earlier in their career would be more likely to be rotating at a large hospital and therefore able to attend the sessions and complete the survey. We have a good distribution of those < 6 years and ≥ 6 years in practice. However, there was a substantial proportion of missing data (*N* = 80; 28%) for years practicing, which could have biased the results.

Mexicali, Mexico, is experiencing a high disease burden due to RMSF, and early diagnosis and treatment are critical to reducing morbidity and mortality. Healthcare workers are aware of the risk, but a further emphasis on treatment recommendations, especially for children, is needed. Further expanding education for healthcare workers on where to find diagnostic testing will also provide valuable information for surveillance and risk assessment in this community.

## Supplemental files


Supplemental materials
