# Supplementary material for: Knowledge, Attitudes, and Practices on Rocky Mountain Spotted Fever among Physicians in a Highly Endemic Region—Mexicali, Mexico
Source: Am J Trop Med Hyg. 2022 Aug 22;107(4):773–9. doi: 10.4269/ajtmh.21-1017 (PMC9651539; doi:10.4269/ajtmh.21-1017)
Supplement: Supplementary file 1 [file tpmd211017.SD1.pdf]

APPENDIX 1: English translation of KAP survey given to healthcare providers in Mexicali, Mexico

Rocky Mountain Spotted Fever Survey

Survey ID#:_________
Date: __/__/____

The purpose of this survey is to gauge current knowledge, attitudes, and practices among clinicians and other health-care providers about tickborne diseases, and Rocky Mountain Spotted Fever (RMSF) in particular.

This survey should take no longer than 10 minutes to complete, and all responses will be anonymous. Results will be used to improve communication and guidance to healthcare providers about RMSF and other tickborne illnesses. There will be no negative consequences if you choose not to participate.

If you would like more information on how this survey will be used, please contact _______________.

Your participation is greatly appreciated, and is completely voluntary.

General Information

1. What is your age? ☐ <25 ☐ 25-44 ☐ 45-64 ☐ 65+
2. What is your sex? ☐ Male ☐ Female ☐ Prefer not to answer
3. Where is your medical practice located (Part of City)? _________________________
4. What is the setting of your practice? ☐ Hospital ☐ Clinic ☐ Pharmacy ☐ Other:_______
5. What is your medical specialty? ☐ Internal Medicine ☐ Emergency Medicine ☐ Pediatrics ☐ Infectious Diseases ☐ Family Medicine ☐ Other:________
6. How many years have you been practicing medicine? ________________

Rocky Mountain Spotted Fever Survey

Part I – Attitudes and Perceptions

1. How confident are you that you have up-to-date knowledge on RMSF?

Not at all confident	Somewhat confident	Confident	Very confident
◯	◯	◯	◯

2. How confident do you feel in diagnosing a patient with RMSF, based on clinical symptoms alone?

◯ ◯ ◯ ◯

3. How confident are you in your knowledge of what type (eg. PCR) of testing to perform for a patient in whom you suspect RMSF.

◯ ◯ ◯ ◯

4. How confident are you in your knowledge of where to have diagnostic testing performed for a patient in whom you suspect RMSF.

◯ ◯ ◯ ◯

4a. If you did not answer "very confident" to 4, why?

☐ I am not sure where testing can be performed

☐ I am not sure how to order the correct testing

☐ I am not sure how to get the results

☐ Other. please specify:_______________

5. I believe that tickborne disease poses a serious risk to individuals in my community.

Strongly disagree	Disagree	Neither agree nor disagree	Agree	Strongly agree
◯	◯	◯	◯	◯

Part II – Individual Practices

6. If a patient presents with a febrile illness, how often do you:

	Rarely	Sometimes	Frequently	Always	NA
Ask if the person has had a tick bite in the past 2 weeks	◯	◯	◯	◯	◯
Ask about ticks in the home or on pets	◯	◯	◯	◯	◯
Inquire about dog ownership	◯	◯	◯	◯	◯
Order one serology test for RMSF	◯	◯	◯	◯	◯
Order PCR to test for RMSF	◯	◯	◯	◯	◯

7. If a patient asks about RMSF or other tickborne illness, how often do you:

	Rarely	Sometimes	Frequently	Always	NA
Provide the patient with educational materials	◯	◯	◯	◯	◯
Discuss risk factors	◯	◯	◯	◯	◯
Discuss preventative measures	◯	◯	◯	◯	◯

8. Have you diagnosed a case of RMSF in the last year?

☐ Yes

☐ No

8a. If yes to 8, how many have you diagnosed in the past year?

____

8b. If yes to 8, how were most diagnosed?

☐ Clinically, without confirmatory lab testing

☐ Clinically, with positive PCR

☐ Clinically, with positive serology

☐ Other, please specify:_______________________

9. If you diagnose a case of RMSF, how often do you:

	Rarely	Sometimes	Frequently	Always	NA
Report it to the local jurisdiction	◯	◯	◯	◯	◯

Part III - Knowledge

10. What percent of patients with RMSF report a history of tick bite?

☐ 90%

☐ 50%

☐ 25%

11. What is the incubation period (time from tick bite to onset of symptoms) for RMSF?

☐ 24-36 hours

☐ 3-14 days

☐ 14-21 days

12. Select the correct statements regarding rash in RMSF (select all that apply):

☐ Treatment should not be started until the rash develops

☐ Rash typically develops between days 2-5 of illness

☐ Rash develops in approximately 90% of people, but may appear after the 5th day of illness

13. Antibiotic therapy should be initiated (choose the best answer):

☐ As soon as the diagnosis of RMSF is suspected

☐ After laboratory confirmation of infection has been obtained

☐ After appearance of a rash

☐ None of the above

14. The antibiotic of choice for treatment of RMSF in adults and children > 8 years old is:

☐ Azithromycin

☐ Chloramphenicol

☐ Doxycycline

☐ Trimethoprim-sulfamethoxazole

15. The antibiotic of choice for treatment of RMSF in children ≤ 8 is:

☐ Azithromycin

☐ Chloramphenicol

☐ Doxycycline

☐ Trimethoprim-sulfamethoxazole

17. How commonly do the following symptoms present in cases of RMSF:

	Always	Frequently	Sometimes	Rarely	Never
Fever	○	○	○	○	○
Headache	○	○	○	○	○
Rash	○	○	○	○	○
Myalgia	○	○	○	○	○
Abdominal pain	○	○	○	○	○
Cough	○	○	○	○	○

18. The fatality rate of untreated RMSF is

☐ Zero

☐ <10%

☐ 20-25%

☐ >50%

19. Which of the following severe manifestations are associated with RMSF (select all that apply):

☐ Gangrene requiring amputation

☐ Cerebral edema and altered mental status

☐ Severe thrombocytopenia

☐ Pulmonary edema and Acute Respiratory Distress Syndrome (ARDS)

20. Over what period of time do the sequela listed in #17 manifest:

☐ 5-7 days after symptom onset

☐ >10 days after symptom onset

☐ These are very rare, and timing is not well established

16. When is therapy for RMSF most effective?

☐ Within 5 days of symptom onset

☐ Within 7-10 days of symptom onset

☐ Within 2 weeks of symptom onset

☐ Timing of antibiotic therapy does not matter

21. Current recommendations to prevent RMSF include (choose all that apply)

☐ Monitor closely for fever, or other symptoms for two weeks following a known tick bite, seek medical attention if symptoms develop

☐ Avoiding outdoor activities and limiting dog ownership

☐ Careful inspection for, detection of, and removal of ticks from pets or persons before or soon after they attach

☐ Use of tick repellent when tick exposure is a possibility, and use of tick repellent or tick-killing substances on pets

☐ Applying to pesticide to yards and homes
